# Characteristics of a cost-effective blood test for colorectal cancer screening

**DOI:** 10.1093/jnci/djae124

**Published:** 2024-06-06

**Authors:** Pedro Nascimento de Lima, Rosita van den Puttelaar, Amy B Knudsen, Anne I Hahn, Karen M Kuntz, Jonathan Ozik, Nicholson Collier, Fernando Alarid-Escudero, Ann G Zauber, John M Inadomi, Iris Lansdorp-Vogelaar, Carolyn M Rutter

**Affiliations:** Engineering and Applied Sciences Department, RAND, Arlington, VA, USA; Department of Public Health, Erasmus Medical Center, Erasmus University, Rotterdam, The Netherlands; Institute for Technology Assessment, Department of Radiology, Massachusetts General Hospital, Boston, MA, USA; Department of Epidemiology and Biostatistics, Memorial Sloan Kettering Cancer Center, New York, NY, USA; Division of Health Policy and Management, University of Minnesota School of Public Health, Minneapolis, MN, USA; Decision and Infrastructure Sciences Division, Argonne National Laboratory, Lemont, IL, USA; Decision and Infrastructure Sciences Division, Argonne National Laboratory, Lemont, IL, USA; Department of Health Policy, School of Medicine, Stanford Health Policy, Freeman-Spogli Institute for International Studies, Stanford University, Stanford, CA, USA; Department of Epidemiology and Biostatistics, Memorial Sloan Kettering Cancer Center, New York, NY, USA; Department of Internal Medicine, University of Utah School of Medicine, Salt Lake City, UT, USA; Department of Public Health, Erasmus Medical Center, Erasmus University, Rotterdam, The Netherlands; Hutchinson Institute for Cancer Outcomes Research and Biostatistics Program, Public Health Sciences Division, Fred Hutchinson Cancer Research Center, Seattle, WA, USA

## Abstract

**Background:**

Blood-based biomarker tests can potentially change the landscape of colorectal cancer (CRC) screening. We characterize the conditions under which blood test screening would be as effective and cost-effective as annual fecal immunochemical testing or decennial colonoscopy.

**Methods:**

We used the 3 Cancer Information and Surveillance Modeling Network–Colon models to compare scenarios of no screening, annual fecal immunochemical testing, decennial colonoscopy, and a blood test meeting Centers for Medicare & Medicaid (CMS) coverage criteria (74% CRC sensitivity and 90% specificity). We varied the sensitivity to detect CRC (74%-92%), advanced adenomas (10%-50%), screening interval (1-3 years), and test cost ($25-$500). Primary outcomes included quality-adjusted life-years (QALY) gained from screening and costs for a US average-risk cohort of individuals aged 45 years.

**Results:**

Annual fecal immunochemical testing yielded 125-163 QALY gained per 1000 at a cost of $3811-$5384 per person, whereas colonoscopy yielded 132-177 QALY gained at a cost of $5375-$7031 per person. A blood test with 92% CRC sensitivity and 50% advanced adenoma sensitivity yielded 117-162 QALY gained if used every 3 years and 133-173 QALY gained if used every year but would not be cost-effective if priced above $125 per test. If used every 3 years, a $500 blood test only meeting CMS coverage criteria yielded 83-116 QALY gained at a cost of $8559-$9413 per person.

**Conclusion:**

Blood tests that only meet CMS coverage requirements should not be recommended to patients who would otherwise undergo screening by colonoscopy or fecal immunochemical testing because of lower benefit. Blood tests need higher advanced adenoma sensitivity (above 40%) and lower costs (below $125) to be cost-effective.

Colorectal cancer (CRC) is the third most common cause of cancer-related mortality among men and women in the United States (1,2). In the year 2023, approximately 153 020 individuals will be diagnosed with CRC, and 52 550 will die from the disease. Screening has reduced the burden of CRC (3,4). The US Preventive Services Taskforce (USPSTF) recommends that average-risk individuals begin CRC screening at age 45 years and endorses a range of screening modalities, including decennial screening colonoscopy, annual fecal immunochemical chemical test, and multitarget stool DNA (mt-sDNA) tests every 3 years (5). Colonoscopy is a screening and diagnostic test, with high sensitivity for detection of cancer and precancerous lesions (6). In the United States, colonoscopy is the most commonly used CRC screening test (63.9%), followed by stool-based tests (7). Yet adherence to CRC screening recommendations is suboptimal; in 2020, 72% of individuals aged 50 years and older were up to date with recommended CRC screening (8). Ongoing adherence to fecal immunochemical testing screening is also low; less than 25% of people participating in fecal immunochemical testing screening were screened annually over a 3-year period (9).

Recently, blood-based biomarkers to detect CRC have emerged as a potential screening modality, and multiple trials are underway to establish the performance of novel blood-based biomarker tests for CRC screening (10). Blood tests are appealing because they are minimally invasive and easily performed in a doctor’s office with minimal risk (11). The Centers for Medicare & Medicaid Services (CMS) released a coverage decision for CRC screening of individuals at average risk for CRC, which would reimburse a triennial blood test with a demonstrated sensitivity of at least 74% for detection of CRC and specificity of at least 90% (12). However, the current CMS coverage decision does not stipulate requirements for adenoma detection, particularly the detection of advanced adenomas that put a person at higher risk of CRC. Moreover, modeling studies project blood tests that just achieve the minimum requirements in CMS’s coverage decision to yield substantially fewer life-years gained than either annual fecal immunochemical testing or decennial colonoscopy (13,14); hence, they could harm those who would otherwise receive annual fecal immunochemical testing or decennial colonoscopy screening.

We performed a threshold analysis of test performance characteristics, interval, and costs using the 3 Cancer Information and Surveillance Modeling Network (CISNET)–CRC microsimulation models to characterize the conditions under which a blood test for CRC screening would be noninferior to the most common US screening regimens, decennial colonoscopy, or annual fecal immunochemical testing, on the basis of effectiveness and cost-effectiveness. We identify characteristics that would render blood tests as effective and cost-effective as the alternatives and how far blood tests would be from the cost-effectiveness frontier under multiple scenarios using the net monetary benefit measure.

## Methods

### Microsimulation models

Analyses used the 3 CRC models part of the CISNET-Colon group: the Microsimulation Screening Analysis-Colon model (MISCAN-Colon) (15,16), the Simulation Model of Colorectal Cancer (SimCRC) (17), and the Colorectal Cancer Simulated Population model for Incidence and Natural history (CRC-SPIN) model (18). These models have been used to inform screening recommendations by the USPSTF (17,19), and analyses used the same assumptions as those made for the 2021 USPSTF analysis (17), including simulation of recent increases in CRC incidence before age 50 years in the US population. For these models, advanced adenomas are defined as adenomas that are 10 mm or larger. More details about the models’ assumptions are available on the CISNET models profiles website (20).

### Screening strategies

Each modeling group simulated a cohort of 10 million individuals from 45 years to death. Four screening strategies were included in base-case scenarios: no screening (used as a comparator), colonoscopy every 10 years and fecal immunochemical testing every year (2 strategies considered cost-effective in prior studies) (21,22), and a blood test every 3 years (following the current CMS coverage decision). We simulated screening from ages 45 to 75 years for all tests. We assumed that all individuals who received an abnormal noninvasive test (fecal immunochemical testing or blood) underwent follow-up colonoscopy. Those with a negative follow-up colonoscopy after a false-positive triage test resumed their original screening regimen 10 years after their colonoscopy. Individuals simulated to have adenomas detected at colonoscopy were assumed to follow surveillance guidelines until age 85 years (23). Individuals were assumed to fully adhere to screening, follow-up colonoscopy, and surveillance.

### Test characteristics

Fecal immunochemical testing characteristics were set at values used in a prior modeling study for the USPSTF (17), whereas colonoscopy sensitivities were updated following a meta-analysis (24) (Supplementary Table 1, available online). Baseline performance characteristics for a blood test were based on the current CMS coverage decision requirements, namely, 74% CRC sensitivity and 90% specificity (12). CMS did not specify advanced adenoma sensitivity requirements; we therefore assumed 10% sensitivity to advanced adenoma as baseline (ie, advanced adenomas are found only because of lack of specificity).

### Economic assumptions

Supplementary Table 2 (available online) contains the disutility and cost assumptions used in this study. Base-case analyses assumed a cost of $500 per blood test. Following a comparable cost-effectiveness analysis (21), we assumed no differential disutility from blood tests relative to fecal immunochemical testing, implying no differential loss in quality of life caused by the 2 tests. Disutility from screening tests, treatments, and screening complications were discounted from the benefit of screening. Costs included screening and treatment and were inflated to 2021 US dollars. Diagnostic colonoscopy costs were incurred by those who tested positive using noninvasive tests and those with symptom-detected CRC (Supplementary Table 2, available online). All costs and benefits were discounted at 3% per year.

### Outcomes

The primary measures of effectiveness were life-years gained and quality-adjusted life-years (QALY) gained relative to no screening over the remaining lifetime of the simulated cohort. We computed the costs of CRC care (including treatment and screening) and net costs relative to no screening. We report the incremental cost-effectiveness ratio (ICER) for nondominated strategies. A strategy is nondominated (ie, efficient) if no other strategy (or combination of strategies) provides more QALY gained at a lower cost (25). We also report the net monetary benefit for all screening strategies relative to no screening using a willingness to pay of $100 000 per QALY gained—a benchmark widely used in the health technology assessment cost-effectiveness (CEA) literature (26). Unlike the ICER, the net monetary benefit measure (net monetary benefit = willingness to pay * QALY gained—net costs) can be used to rank all strategies on a single scale (27), which is useful in this analysis because blood-based tests and other novel tests were projected to not be cost-effective in prior analyses (21,22). The net monetary benefit of a screening strategy can be interpreted as the dollar value of screening accrued to society and is useful for comparing the value provided by novel CRC screening tests relative to the standard of care of CRC screening (ie, decennial colonoscopy and annual fecal immunochemical testing).

### Threshold analyses

We developed a full factorial experimental design consisting of 900 scenarios to explore the conditions under which blood tests are projected to be noninferior to fecal immunochemical testing or colonoscopy on the basis of effectiveness and cost-effectiveness. Effectiveness noninferiority requires that the test provides at least the same benefit (QALY gained) as the comparator (ie, colonoscopy). Cost-effectiveness noninferiority is the same as nondominance. In this analysis, we varied 4 test characteristics: blood test sensitivity for CRC (74%, 83%, and 92%), sensitivity for advanced adenomas (10%, 20%, 30%, 40%, and 50%), screening interval (1, 2, and 3 years), and test cost ($25-$500 in increments of $25). These values were selected to cover the plausible characteristics of blood tests that will be developed in the near future. The range of CRC sensitivity spans the current CMS minimum threshold (74%) up to the sensitivity of the currently available mt-sDNA tests (92%) (28) and encompasses the CRC sensitivity of existing blood-based biomarker tests, such as the Shield test (83%) (29). Sensitivity to detect advanced adenomas spans the false-positive rate under the CMS coverage decision (10%), and the maximum sensitivity to advanced adenomas explored (50%) goes beyond the advanced adenoma sensitivity of any existing noninvasive test: sensitivity to advanced adenomas for mt-sDNA was 42% for the first-generation test (28), 43.4% for the next-generation mt-sDNA test (30), and 46% for a multitarget stool RNA test (31). We also examined shorter screening intervals than the current CMS coverage decision. Finally, we reduced the price of CRC blood tests from the assumed baseline price of approximately $500 (32), down to $25—the approximate price of fecal immunochemical testing tests (Supplementary Table 2, available online).

The specificity of blood tests was held at 90% across all analyses, under the expectation that blood test developers will set detection thresholds as to maximize sensitivity subject to CMS’s minimum specificity constraint. Importantly, the CMS coverage decision specified a minimum CRC specificity of 90% but did not mention precursor lesions, making it unclear whether adenoma detection in the absence of CRC is to be considered a false-positive. Consistent with assumptions of prior modeling studies (17,18), we assume that a false-positive blood test only occurs when neither CRC nor adenomas are detected at colonoscopy.

We evaluated the cost-effectiveness of blood test screening regimens for each of the 900 scenarios (unique combinations of blood test accuracy, costs, and test interval) for each of the 3 models. We identified the scenarios in which blood tests were not dominated by either colonoscopy or fecal immunochemical testing. We report the scenarios that would render blood testing to be considered cost-effective by at least 1 of the 3 models (with an ICER under $150 000 per QALY gained—the upper range of willingness to pay values commonly used in cost-effectiveness analyses) (26).

### Scenario discovery

Because of the large number of scenarios considered, a strategy was needed to 1) identify the test characteristics that have the strongest effect on the cost-effectiveness of blood tests and 2) present a low-dimensional summary of our results to describe how far a blood test scenario is from the cost-effectiveness frontier as a function of test characteristics. We drew from the robust decision making (33,34) approach to accomplish both tasks. In particular, we use the third step in the method, called *scenario discovery* (35), to identify conditions under which blood test screening regimens would fail or succeed in matching or exceeding the net monetary benefit of colonoscopy (the most used screening method in the United States) without requiring a probabilistic characterization of the test characteristics varied.

To identify test characteristics with the biggest effect on the cost-effectiveness of a blood test, we trained a random forest on the predicted cost-effectiveness status (ie, cost-effective vs not cost-effective relative to colonoscopy and fecal immunochemical testing) for each scenario using 5 independent variables: the 4 test characteristics (CRC and advanced adenoma sensitivity, screening interval, and cost) and the natural history model. The goal of this step is to ensure that we select and present the most relevant test characteristics in our analysis. We then used the random forest’s ranking of variable importance, using the mean decrease in Gini coefficient (36,37), to identify the test characteristics most strongly associated with cost-effectiveness.

Next, we chose the 3 variables with the largest effect on the cost-effectiveness of a blood test and included these as independent variables in a locally weighted regression model (38) to generate a smooth approximation of the net monetary benefit of screening given the 3 independent variables in an evenly spaced 3-dimensional grid of 10 000 points. These projections are conditioned on a CRC sensitivity of 92% (the upper range of the threshold analysis) and are averaged across the 3 models. We created those projections for the upper range of CRC sensitivity because it was the least important variable in our experimental design to determine the cost-effectiveness of blood tests and because this is a best-case scenario for finding the blood tests to be cost-effective; hence, it helps reveal the maximum cost and minimum advanced adenoma sensitivity at which blood tests provide equivalent net monetary benefit relative to colonoscopy if blood tests achieve 92% CRC sensitivity. Finally, we computed contour lines on the basis of the smoothed net monetary benefit estimates. This approach produces a “map” that test developers and policy makers can use to assess the net monetary benefit provided by new blood tests or other noninvasive tests with test performance in the range explored in this study in the context of the net monetary benefit of colonoscopy, the leading US alternative.

## Results

### Base case

In the absence of screening, the models projected 32-36 CRC deaths over the remaining lifetime of 1000 individuals aged 45 years (Table 1) and an average cost of $5268-$5845 per person for CRC care. Under a program of annual fecal immunochemical testing screening, models projected 6-12 CRC deaths per 1000 individuals aged 45 years, yielding 125-163 QALY gained per 1000 individuals relative to no screening. Annual fecal immunochemical testing resulted in a cost of $3811-$5384 per person, including both CRC screening and treatment costs, making it a cost-saving strategy relative to no screening. Colonoscopy screening further reduced CRC deaths to 4-10, yielding 132-177 QALY gained per 1000 individuals relative to no screening at a cost of $5375-$7031 per person. The ICER of decennial colonoscopy relative to annual fecal immunochemical testing was $100 125-$250 971 per QALY gained. The net monetary benefit of both strategies was similar, valued at $12 763-$18 128 per person for fecal immunochemical testing and $11 773-$18 126 for colonoscopy at a $100 000 willingness to pay per QALY gained.

**Table 1.** Cost-effectiveness results for comparator tests and select blood tests

Scenario class	Test, interval	Test cost^a^	Sensitivity^b^		CRC deaths^c^	QALY gained^c^^,^^d^	Costs ($)^c^^,^^d^	Net monetary benefit ($) ^e^	ICER ($)^f^	
			Advanced adenoma	CRC						
Base case	No screening	^—^ ^h^	^—^ ^h^	^—^ ^h^	32-36	0	5268-5845	0	Dominated	^—^ ^g^
	Fecal immunochemical testing, 1 y	23.42	0.238	0.738	6-12	125-163	3811-5384	12 763-18 128	Reference strategy	^M, C, S^
	Colonoscopy, 10 y	963.95-1312.36^i^	0.91	0.91	4-10	132-177	5375-7031	11 773-18 126	100 125-250 971	^M, C, S^
	Blood, 3 y	500	0.1	0.74	14-18	83-116	8559-9413	4853-8657	Dominated	^—^ ^g^
No change in blood test cost	Blood, 3 y	500	0.1	0.92	13-17	86-125	8636-9410	5251-9578	Dominated	^—^ ^g^
	Blood, 3 y	500	0.5	0.74	7-13	113-158	6850-8455	8516-14 619	Dominated	^—^ ^g^
	Blood, 3 y	500	0.5	0.92	6-13	117-162	6868-8456	8876-15 044	Dominated	^—^ ^g^ ^-^
	Blood, 1 y	500	0.1	0.74	7-12	119-154	11 282-12 634	4873-9593	Dominated	^—^ ^g^
	Blood, 1 y	500	0.5	0.92	4-10	133-173	10 024-11 560	7388-13 022	Dominated	^—^ ^g^
3 models project blood test as cost-effective^j^	Blood, 1 y	50	0.4	0.83	5-10	131-171	4602-6207	12 515-18 244	53 740-143 193	^M, C, S^
	Blood, 1 y	50	0.4	0.92	5-10	131-172	4602-6211	12 586-18 301	61 830-127 195	^M, C, S^
	Blood, 1 y	50	0.5	0.74	4-10	132-172	4548-6139	12 660-18 425	61 472-115 743	^M, C, S^
	Blood, 1 y	50	0.5	0.83	4-10	132-173	4547-6133	12 743-18 484	63 510-95 272	^M, C, S^
	Blood, 1 y	50	0.5	0.92	4-10	133-173	4549-6132	12 816-18 534	67 399-112 977	^M, C, S^

a Centers for Medicare & Medicaid Services (CMS) cost per covered screening test (applied to those aged older than 65 years). Costs for blood tests, which are not currently covered by Centers for Medicare & Medicaid Services, were assumed to be similar to the cost of Epi proColon.^32^ See Supplementary Table 2 (available online) for detailed costs. CRC = colorectal cancer; CRCSPIN = Colorectal Cancer Simulated Population model for Incidence and Natural history; MISCAN = Microsimulation Screening Analysis model; QALY = quality-adjusted life-years; SIMCRC = Simulation Model of Colorectal Cancer.

b Sensitivity of colonoscopy refers to lesion-level sensitivity, whereas sensitivity for noninvasive tests refers to person-level sensitivity, which considers the most advanced lesion of a screened individual.

c Outcomes presented as events or life-years per 1000 individuals aged 45 years.

d Discounted at 3% per year.

e Net monetary benefit per person setting a willingness to pay of $100 000 per QALY gained (ie, net monetary benefit = 100 000 * QALY gained—net costs). Net costs are the cost of the screening regimen minus the costs of the no screening regimen. In this context, the net monetary benefit can be interpreted as the average absolute monetary value for those who adhere to a screening regimen, accounting for all benefits and costs incorporated in the analysis. The difference between the net monetary benefit of the 2 strategies reveals the loss of benefit caused by choosing a suboptimal strategy.

f Because no screening is a dominated strategy, incremental cost-effectiveness ratio (ICER) is not calculated for the least effective nondominated strategy. M, C, and S refer to MISCAN, CRCSPIN, and SIMCRC and are displayed if each of those models projects the test to be cost-effective (ie, strategy was not dominated and had an ICER lower than $150 000). ICER ranges reflect the range of ICERs for the models that projected a strategy not to be extended dominated.

g ICER is not computed by any model because the screening strategy is dominated.

h The no screening strategy has no test cost or sensitivity values.

i Screening colonoscopy costs depend on whether lesions are removed during the exam. We provide the lower cost, without lesion removal, and the higher cost, with lesion removal. Further detail is provided in Supplementary Table 2 (available online).

j This table presents only blood tests projected as cost-effective by the 3 models at a $50 cost. Supplementary Table 3 (available online) contains other combinations of test performance characteristics where 2 or 3 models project blood tests to be cost-effective.

A blood test with the minimum performance criteria put forth by CMS would be more costly and less effective than either decennial colonoscopy or annual fecal immunochemical testing. If used every 3 years, a blood test meeting these minimal performance characteristics would result in 14-18 CRC deaths and 83-116 QALY gained per 1000 individuals at a total cost of $8559-$9413 per person. Although the net monetary benefit of the test is positive at $4853-$8657, making the test cost-effective relative to no screening, this triennial test regimen would yield 34%-50% fewer QALY gained relative to decennial colonoscopy, resulting in a loss of 52%-70% of the net monetary benefit provided by decennial colonoscopy.

### Threshold analyses

#### Effectiveness thresholds

**Figure 1.**
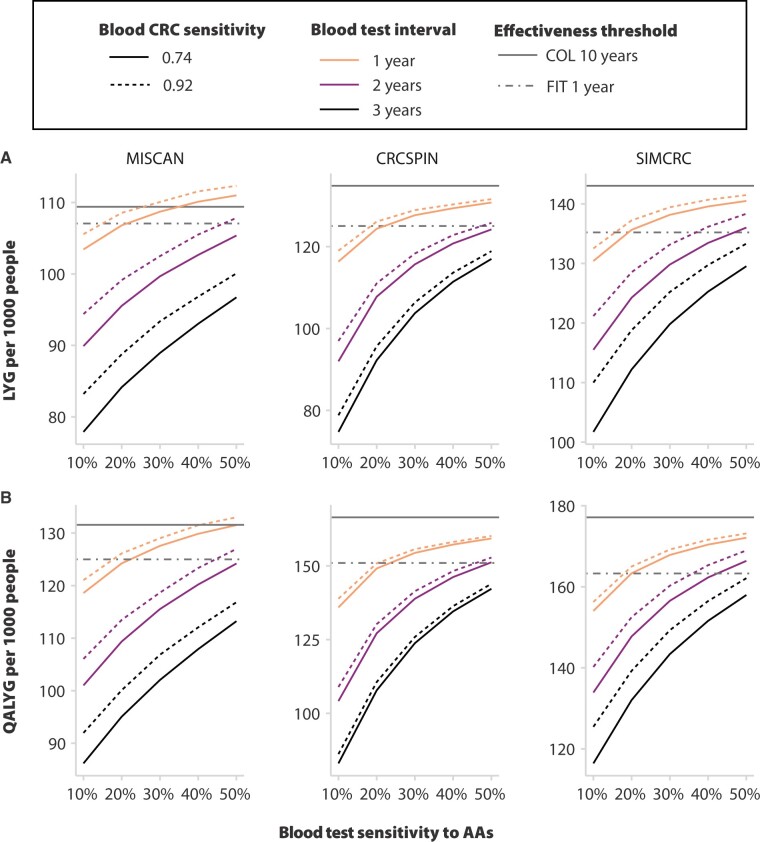
Effectiveness of blood colorectal cancer (CRC) screening tests as a function of sensitivity and interval. Each **curve** represents projected lifetime benefits per 1000 guideline-adherent individuals aged 45 years for alternative blood-based screening regimens as a function of blood tests’ sensitivity to CRC and advanced adenomas. **Horizontal lines** represent the projected benefits of annual fecal immunochemical testing and decennial colonoscopy. Noninvasive tests’ sensitivity is modeled as sensitivity to the most advanced lesion in the colon. Outcomes are discounted at 3% per year. All screening regimens start at 45 years and end at 75 years. **Panel A** presents discounted life-years gained from screening per 1000 individuals aged 45 years. **Panel B** presents quality-adjusted life-years gained from screening per 1000 individuals aged 45 years. **Vertical axes** vary across models to facilitate visualization of relative differences. AA = advance adenoma; COL = colonoscopy; FIT = fecal immunochemical testing; LYG = life-years gained; QALYG = quality-adjusted life-years gained.

A blood test with a 1- or 2-year screening interval and relatively high advanced adenoma sensitivity could be noninferior to annual fecal immunochemical testing in terms of life-years gained (Figure 1 A) and QALY gained (Figure 1 B) for some combinations of interval and advanced adenoma sensitivity. For instance, an annual blood test regimen with 74% CRC sensitivity and 20% advanced adenoma sensitivity was noninferior to annual fecal immunochemical testing in terms of QALY gained, but a biennial blood test regimen with 74% CRC sensitivity would require nearly 50% advanced adenoma sensitivity to be noninferior to fecal immunochemical testing. A triennial blood test regimen with the best accuracy assumptions (92% CRC sensitivity and 50% advanced adenoma sensitivity) nearly achieved QALY gained noninferiority, yielding 117-162 QALY gained per 1000 individuals, but models still projected a 1%-7% effectiveness gap relative to fecal immunochemical testing and an 8%-14% QALY gained gap relative to colonoscopy. Increasing a blood test’s sensitivity to advanced adenomas from 10% to 20% increased effectiveness more than increasing CRC sensitivity from 74% to 92% across the 3 models, but the marginal benefit of advanced adenoma sensitivity improvements decreases at higher sensitivity levels. Of the 3 models, 2 (CRCSPIN and SimCRC) projected that blood test screening strategies provided inferior QALY gained relative to decennial colonoscopy in all scenarios.

#### Cost-effectiveness thresholds

**Figure 2.**
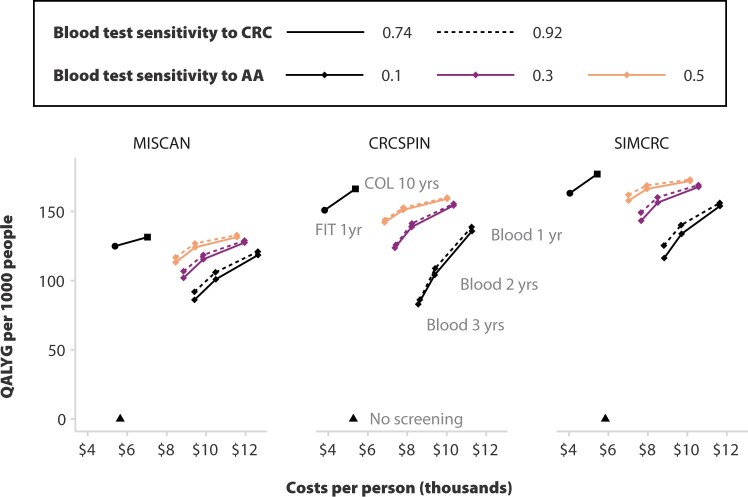
Cost-effectiveness frontier for blood tests with varying test performance. The **triangle**, **circle**, and **square** shapes represent the projected performance of no screening, annual fecal immunochemical testing, and decennial colonoscopy in the cost-effectiveness plane. The **line** connecting fecal immunochemical testing and colonoscopy is the cost-effectiveness frontier (ie, the line connecting all strategies that provide good value for money). **Diamond** shapes connected by lines represent the performance of blood testing with varying levels of colorectal cancer (CRC) and advanced adenoma sensitivity, with **solid lines** representing the lower end of the simulated CRC sensitivity range (0.74) and **dashed lines** representing the higher end of the simulated CRC sensitivity range (0.92). Separate panels present projections from each of the 3 Cancer Information and Surveillance Modeling Network models. AA = advanced adenoma; COL = colonoscopy; FIT = fecal immunochemical testing; QALYG = quality-adjusted life-years gained.

None of the models projected that blood testing would be cost-effective without substantial reductions in price and higher sensitivity to advanced adenomas (Figure 2, Table 1). An annual blood test regimen with 50% advanced adenoma sensitivity and 92% CRC sensitivity was noninferior in effectiveness relative to annual fecal immunochemical testing. However, it would cost $10 024-$11 560 per person for a $500 blood test—well above the costs of either fecal immunochemical testing ($3811-$5384 per person) or decennial colonoscopy ($5375-$7031 per person) without yielding higher benefit.

The conditions under which a blood test would be cost-effective varied across models (Table 1). For example, all models projected that a $50 blood test with 50% advanced adenoma sensitivity and 92% CRC would be cost-effective, yielding 133-173 QALY gained at a cost of $4549-$6132 per person. Such a test would yield an ICER of $67 399-$112 977 per QALY gained. Table 1 provides other combinations of performance characteristics of a $50 blood test for which all 3 models projected blood testing to be nondominated and cost-effective with a willingness to pay under $150 000. There were also scenarios where only 1 or 2 models projected blood testing to be cost-effective (Supplementary Table 3, available online). The maximum unit cost at which blood testing was cost-effective for at least 1 of the models (MISCAN) was $125. At this price, the test would need above 50% advanced adenoma sensitivity, above 83% CRC sensitivity, and to be used annually. Conversely, a test with 30% advanced adenoma sensitivity was only cost-effective per 2 models if priced at $25 per test. Supplementary Table 3 (available online) provides additional outcomes (life-years gained and CRC cases) along with the full set of 44 scenarios for which 1, 2, or the 3 models project blood tests to be cost-effective.

#### Net monetary benefit non-inferiority scenarios

**Figure 3.**
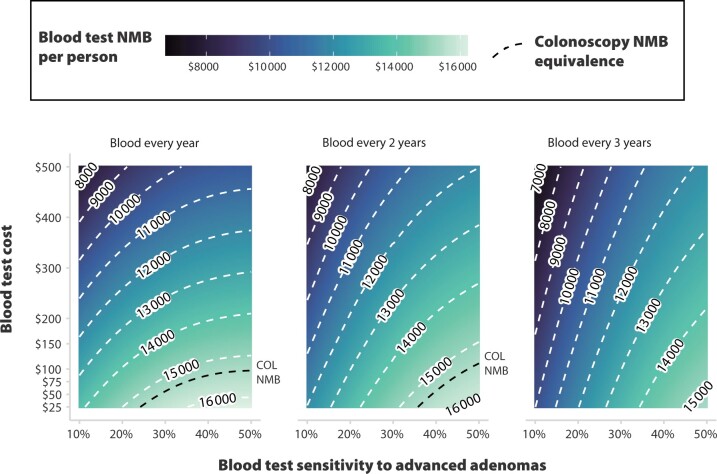
Blood test net monetary benefit as a function of performance, interval, and costs. Colors estimate the average net monetary benefit of blood tests across models as a function of test (Centers for Medicare & Medicaid Services) cost, sensitivity to advanced adenomas, and test interval. Colorectal cancer (CRC) sensitivity is set at 92% (upper range of the experimental design). Each panel represents 1 blood-based screening interval. **White dashed contour lines** represent parameter combinations at which the net monetary benefit is constant, and the **black dashed line** represents the average net monetary benefit of decennial colonoscopy across the 3 models ($15 479 per person). This figure can be used to identify conditions under which blood tests provide equal or superior net monetary benefit relative to decennial colonoscopy (ie, the area under the **black dashed line**) and to estimate the gap between the net monetary benefit provided by blood-based tests and colonoscopy. For example, a blood-based test that costs $300 and has 10% advanced adenoma sensitivity yields a $10 000 net monetary benefit, which is approximately two-thirds of the net monetary benefit of colonoscopy. Further, a $500 blood test that is used every 3 years will still be approximately $3000 (ie, 20%) short of matching the net monetary benefit of colonoscopy even if it has 50% advanced adenoma sensitivity and 92% CRC sensitivity. For reference, the average net monetary benefit of annual fecal immunochemical testing estimated by the models is $15 815. COL = colonoscopy; NMB = net monetary benefit.

The random forest trained to predict the cost-effectiveness of blood screening strategies relative to colonoscopy resulted in the following ranking of variable importance: 1) test cost, 2) advanced adenoma sensitivity, 3) test interval, 4) model, and 5) CRC sensitivity, confirming the small relative contribution of improved CRC sensitivity vs higher advanced adenoma sensitivity over the range we explored. Figure 3 shows the net monetary benefit for blood test screening regimens as a function of the 3 most important test characteristic inputs (test cost, advanced adenoma sensitivity, and test interval), averaged across the 3 models and assuming tests have 92% CRC sensitivity. A $500 test with 10% advanced adenoma sensitivity provided inferior net monetary benefit compared with colonoscopy, regardless of the screening frequency. Across the 3 interval scenarios, the test did not provide more than $8000 of net monetary benefit, whereas colonoscopy was estimated to provide $15 400 of net monetary benefit. Second, if a blood test were to achieve advanced adenoma sensitivity of 40%-50%, but cost $500 per test, blood testing would still fall approximately 20% short of matching the net monetary benefit of colonoscopy. Finally, if blood tests were offered at a cost lower than $125 per test and were to achieve advanced adenoma sensitivity of 40% or higher, the net monetary benefit of blood testing could be above $14 000, closing the net monetary benefit gap to approximately $1000 (or 7% of the net monetary benefit of colonoscopy).

## Discussion

Blood-based biomarkers for CRC screening must have substantially better test accuracy than the CMS coverage decision to achieve effectiveness similar to either annual fecal immunochemical testing or decennial colonoscopy and must cost considerably less to be cost-effective. Multiple combinations of test characteristics may render blood tests cost-effective, but all of them required the cost of the blood test to be $125 or lower and advanced adenoma sensitivity to be 30% or higher, and higher accuracy was needed to justify higher prices. For instance, 50% sensitivity to advanced adenoma was needed to justify a $125 blood test, whereas a blood test with 30% advanced adenoma sensitivity would have to be priced at $25 per test to be cost-effective. This result underscores the value of higher sensitivity to precursor lesions because their detection and removal prevents cancer and reduces health costs associated with cancer care.

Net monetary benefit estimates provide an objective measure of the value of screening to society and can be used to compare alternatives on a single scale. By this metric, if blood tests cost $500, only meet minimum CMS accuracy criteria, and are used every 3 years, their adoption could eliminate 52%-70% of the net monetary benefit afforded by colonoscopy for individuals who would otherwise follow colonoscopy screening. In contrast, annual fecal immunochemical testing’s net monetary benefit is on par with colonoscopy. Although there may be valid reasons to make blood tests available for CRC screening, they are unlikely to justify such a loss of benefit among individuals who would otherwise undergo colonoscopy screening. Beyond quantifying differences in long-term outcomes for different blood test screening regimens and currently recommended fecal immunochemical testing and colonoscopy regimens, this study identifies the characteristics of blood tests that would reduce or eliminate the potential loss caused by the adoption of blood tests. Even if blood tests cannot yet achieve the performance and cost thresholds we identified, this study can be used to quantify the gap between the value afforded by blood tests and colonoscopy.

Our findings align with previously published analyses but provide further information for policy makers and blood test developers. One study found that blood tests that only met the minimum CRC sensitivity from CMS and had low advanced adenoma sensitivity would frequently yield inferior results compared with fecal immunochemical testing and mt-sDNA (39). A second study explored multiple hypothetical tests that met or exceeded the CMS sensitivity and showed that a blood test with higher sensitivity for advanced adenoma would yield better benefits but did not consider cost-effectiveness (40). This analysis varied 4 test characteristics—test interval, sensitivity to CRC, sensitivity to advanced adenomas, and unit price—that developers can aim for with the goal of providing an effective and cost-effective new CRC screening modality. Still, we held several inputs constant in this analysis, including the specificity of the blood test, and we only included annual (vs biennial) fecal immunochemical testing as a comparator. Future research may include a wider range of comparators, including a fecal immunochemical test with a lower detection threshold calibrated to have 90% specificity.

Importantly, we assumed perfect adherence to primary screening and follow-up colonoscopy across the different screening regimens and intervals. A thorough evaluation of imperfect adherence would require considering differential adherence to fecal immunochemical testing and colonoscopy screening and real-world adherence to follow-up colonoscopy. Although data on follow-up colonoscopy adherence are scant, the existing data justify the concern that blood-based CRC screening might entail low adherence to follow-up colonoscopy. A randomized trial of patient adherence found higher adherence rates to screening for blood tests relative to fecal immunochemical testing but lower adherence rates to follow-up colonoscopy (41). Such an analysis would need to factor in trade-offs involved in improving adherence vs reducing the overall effectiveness of the screening program in the population and increasing costs. Finally, our analysis focused on blood tests that target CRC, as opposed to multicancer early detection tests. Future work may expand on our results to inform the adoption of novel blood-based biomarker tests for CRC screening.

## Supplementary Material

djae124_Supplementary_Data

## Data Availability

Data underlying figures and tables will be shared upon request.
