# Supplementary material for: Characteristics of a cost-effective blood test for colorectal cancer screening
Source: J Natl Cancer Inst. 2024 Jun 6;116(10):1612–20. doi: 10.1093/jnci/djae124 (PMC11461157; doi:10.1093/jnci/djae124)
Supplement: djae124_Supplementary_Data [file djae124_supplementary_data.pdf]

Supplementary Material

Supplementary materials for Nascimento de Lima et al. 2024: **"***Cost-effectiveness threshold analysis of blood-based tests for colorectal cancer screening"*

Contents

Supplementary Table 1. Test Performance Assumptions

Supplementary Table 2. Cost and disutility from screening tests, CRC care, and Colonoscopy complications

Supplementary Table 3. Cost-effectiveness results for comparator tests and cost-effective blood tests

Supplementary Table 1. Test Performance Assumptions

Test	1- specificity	CRC sensitivity	Adenoma sensitivity			Source
			1 to <6mm	6 to <10mm	≥10mm	
FIT[a]	0.036	0.738	0.076‡		0.238†	1
Blood test[b]	0.1	0.74	0.1±	0.1±	0.1±	2
Colonoscopy[c]	0.1325§	0.91	0.69	0.81	0.91	3
Threshold analysis						
Blood test[d]	0.1	0.74 - 0.92	0.1 - 0.126	0.1 - 0.26	0.1 - 0.5	-

CRC = colorectal cancer, FIT = fecal immunochemical test.

a) Sensitivity per person based on most advanced lesion.

* Per individual for FIT and blood test, based on most advanced lesion, and per lesion for endoscopy

‡ Sensitivity for persons with non-advanced adenomas. For persons with 1 to <6mm adenomas, MISCAN and SimCRC assumed that the sensitivity of the test is equal to the positivity rate in people without adenomas or cancer (1 – specificity). The sensitivity for persons with adenomas 6 to <10mm was chosen such that the weighted average sensitivity is equal to that for non-advanced adenomas.

†Sensitivity for advanced adenomas (i.e., adenomas ≥ 10mm and/or adenomas with advanced histology). We assume no advanced histology in adenomas <10mm.

± Adenomas are only detected by chance, with sensitivity set to the positivity rate in people without adenomas or cancer (1 – specificity).

§ The lack of specificity with colonoscopy reflects the detection of non-adenomatous lesions that are removed and therefore induce costs due to polypectomy and biopsy.

** Relative sensitivity for stage I vs. stage II-IV. The absolute sensitivity for stage I and stage II-IV values were calculated such that the weighted overall sensitivity for stage I-IV was equal to the base-cases sensitivities of 73.8% and 74% for FIT and blood-based screening, respectively.

Supplementary Table 2. Cost and disutility from screening tests, CRC care and Colonoscopy complications.

A. Screening tests				
**Test**	*Commercial costs*	*CMS costs*	*Disutility when positive*	*Disutility when negative*
**Colonoscopy**[a]				
Screening w/o lesion removal	1,427.67	963.95	NA	0.000496
Diagnostic w/o lesion removal[b]	1,427.67	909.18	NA	0.000496
Surveillance w/o lesion removal	1,427.67	907.53	NA	0.000496
Any colonoscopy with lesion removal	1,889.78	1312.36	0.001401	NA
**FIT**[c]	25.53	23.42	0.00133	0.000063
**Blood-based test**	675*	500[d]	0.00133	0.000063

B. Colorectal cancer care				
**2007-2013 Commercial* costs per LY CRC care**[e]	*Initial care*	*Continuing care*	*Terminal care Death CRC*	*Terminal care Death OC*
Stage I CRC	55,570	78,801	114,491	170,133
Stage II CRC	5,719	6,650	10,289	48,776
Stage III CRC	112,144	126,413	132,346	166,423
Stage IV CRC	29,452	31,693	43,327	104,221
**2007-2013 CMS costs per LY CRC care**[e]				
Stage I CRC	41,163	58,372	84,809	126,025
Stage II CRC	4,236	4,926	7,622	36,130
Stage III CRC	83,069	93,639	98,034	123,308
Stage IV CRC	21,816	23,476	32,094	77,200
**Utility loss per LY with CRC care**[e, f]				
Stage I CRC	0.12	0.05	0.7	0.05
Stage II CRC	0.18	0.05	0.7	0.05
Stage III CRC	0.24	0.24	0.7	0.24
Stage IV CRC	0.7	0.7	0.7	0.7

C. Colonoscopy complications			
Event	*Commercial* costs*	*CMS costs*	*Utility loss*
Serious gastrointestinal event[g]	11,715.00	8,677.80	0.0055
Other gastrointestinal event[h]	8,862.00	6,564.40	0.0027
Cardiovascular event[i]	9,541.60	7,0.67.8	0.0048

CMS = Centers for Medicare and Medicaid Services, FIT = fecal immunochemical test, CRC = colorectal cancer

* For individuals younger than 65, commercial costs were used, whereas for individuals aged 65 years and older, CMS costs were used. As no data are available to inform commercial cost estimates for colonoscopy complications and CRC treatment, CMS rates were multiplied by 1.35 for individuals younger than age 65 years based on the observed mean ratio of commercial to

Medicare payment rates for colorectal tests [4].

a. Colonoscopy costs were based on an analysis of 2014 CMS data and inflated to 2021 USD using the 2021 Personal Health Care Deflator Price Index.

b. Used for colonoscopies performed as a diagnostic follow-up after a positive non-colonoscopy test and for colonoscopies performed to diagnose symptom-detected CRC cases.

c. FIT costs were based on 2021 Clinical Laboratory Fee Schedule data and inflated to 2021 USD using the 2021 Personal Health Care Deflator Price Index.

d. Costs for blood-tests, which are not currently covered by CMS, were assumed to be similar to the cost of Epi proColon®.[5]

e. Care for CRC was divided into three clinically relevant phases: initial, continuing, and terminal care. The initial care phase was defined as the first 12 months after diagnosis; the terminal care phase was defined as the final 12 months of life; the continuing care phase was defined as all months in between. In the terminal care phase, we distinguished between CRC patients dying from CRC and CRC patients dying from another cause. For patients surviving less than 24 months, the final 12 months were allocated to the terminal care phase, and the remaining months were allocated to the initial care phase.

f. Utility losses for life years (LYs) with initial care were derived from a study by Ness et al. [6]. For LYs with continuing care for stage I and II CRC, we assumed a utility loss of 0.05 QALYs; for LYs with continuing care for stage III and IV CRC, we assumed the corresponding utility losses for LYs with initial care. For LYs with terminal care for CRC, we assumed the utility loss for LYs with initial care for stage IV CRC. For LYs with terminal care for another cause, we assumed the corresponding utility losses for LYs with continuing care.

h. Serious gastrointestinal events are perforations, gastrointestinal bleeding, or transfusions. The rate depends on age, formula: 1/[exp(9.27953 - 0.06105 x Age) + 1] - 1/[exp(10.78719 - 0.06105 x Age) + 1].

h. Other gastrointestinal events are paralytic ileus, nausea and vomiting, dehydration, or abdominal pain. The rate depends on age, formula: 1/[exp(8.81404 - 0.05903 x Age) + 1] - 1/[exp(9.61197 - 0.05903 x Age) + 1].

i. Cardiovascular events are myocardial infarction or angina, arrhythmias, congestive heart failure, cardiac or respiratory arrest, syncope, hypotension, or shock. The rate depends on age, formula: 1/[exp(9.09053 - 0.07056 x Age) + 1] - 1/[exp(9.38297 - 0.07056 x Age) + 1].

Supplementary Table 3. **Cost-effectiveness results for comparator tests and all cost-effective blood test performance scenarios.**

Scenario Class	Test, interval	Test Cost[a]	Sensitivity[b] AA	Sensitivity[b] CRC	CRC Deaths[c]	QALYG[c,d]	Costs[c,d]	NMB[e]	ICER[f]	
Base case	No screening	—[h]	—[h]	—[h]	32-36	0	5,268-5,845	0	Dominated	—[g]
	FIT, 1yr	23.42	0.238	0.738	6-12	125-163	3,811-5,384	12,763-18,128	Reference strategy	M, C, S
	COL, 10yrs	—[i]	0.91	0.91	4-10	132-177	5,375-7,031	11,773-18,126	100,125-250,971	M, C, S
	Blood, 3yrs	500	0.1	0.74	14-18	83-116	8,559-9,413	4,853-8,657	Dominated	—[g]
No change in test cost	Blood, 3yrs	500	0.1	0.92	13-17	86-125	8,636-9,410	5,251-9,578	Dominated	—[g]
	Blood, 3yrs	500	0.5	0.74	7-13	113-158	6,850-8,455	8,516-14,619	Dominated	—[g]
	Blood, 3yrs	500	0.5	0.92	6-13	117-162	6,868-8,456	8,876-15,044	Dominated	—[g]
	Blood, 1yr	500	0.1	0.74	7-12	119-154	11,282-12,634	4,873-9,593	Dominated	—[g]
	Blood, 1yr	500	0.5	0.92	4-10	133-173	10,024-11,560	7,388-13,022	Dominated	—[g]
Three models project blood test as cost-effective	Blood, 1yr	50	0.4	0.83	5-10	131-171	4,602-6,207	12,515-18,244	53,740-143,193	M, C, S
	Blood, 1yr	50	0.4	0.92	5-10	131-172	4,602-6,211	12,586-18,301	61,830-127,195	M, C, S
	Blood, 1yr	50	0.5	0.74	4-10	132-172	4,548-6,139	12,660-18,425	61,472-115,743	M, C, S
	Blood, 1yr	50	0.5	0.83	4-10	132-173	4,547-6,133	12,743-18,484	63,510-95,272	M, C, S
	Blood, 1yr	50	0.5	0.92	4-10	133-173	4,549-6,132	12,816-18,534	67,399-112,977	M, C, S
	Blood, 1yr	25	0.4	0.74	5-11	130-170	4,291-5,910	12,724-18,490	49,189-108,040	M, C, S
	Blood, 1yr	25	0.4	0.83	5-10	131-171	4,292-5,900	12,822-18,556	40,070-89,739	M, C, S
	Blood, 1yr	25	0.4	0.92	5-10	131-172	4,293-5,903	12,894-18,613	46,281-79,892	M, C, S
	Blood, 1yr	25	0.5	0.74	4-10	132-172	4,244-5,837	12,962-18,731	52,189-69,470	M, C, S
	Blood, 1yr	25	0.5	0.83	4-10	132-173	4,243-5,832	13,045-18,790	50,055-75,861	M, C, S
	Blood, 1yr	25	0.5	0.92	4-10	133-173	4,245-5,831	13,117-18,840	54,277-90,147	M, C, S
	Blood, 2yrs	25	0.5	0.92	5-11	127-169	3,846-5,475	12,871-18,799	6,050-45,689	M, C, S
	Blood, 1yr	75	0.5	0.83	4-10	132-173	4,851-6,435	12,441-18,178	76,153-76,964	M, C

Scenario Class	Test, interval	Test Cost[a]	Sensitivity[b]		CRC Deaths[c]	QALYG[c,d]	Costs[c,d]	NMB[e]	ICER[f]	
			AA	CRC						
Two models project blood test as cost-effective	Blood, 1yr	75	0.5	0.92	4-10	133-173	4,853-6,434	12,514-18,228	80,522-89,957	M, C
	Blood, 1yr	50	0.4	0.74	5-11	130-170	4,600-6,217	12,417-18,178	92,927-171,219	C, S
	Blood, 1yr	25	0.3	0.83	5-11	128-169	4,373-6,009	12,466-18,185	89,254-190,493	C, S
	Blood, 1yr	25	0.3	0.92	5-11	129-169	4,373-6,007	12,543-18,247	79,882-154,724	C, S
	Blood, 2yrs	50	0.5	0.92	5-11	127-169	4,054-5,684	12,662-18,588	19,054-150,572	C, S
	Blood, 2yrs	25	0.5	0.83	5-11	125-168	3,846-5,486	12,700-18,672	5,372-266,600	C, S
	Blood, 3yrs	25	0.5	0.74	7-13	113-158	3,748-5,424	11,546-17,729	Reference strategy	C, S
	Blood, 3yrs	25	0.5	0.83	7-13	115-160	3,756-5,424	11,756-17,943	Reference strategy	C, S
	Blood, 3yrs	25	0.5	0.92	6-13	117-162	3,766-5,425	11,907-18,154	Reference strategy	C, S
One model project blood test as cost-effective	Blood, 1yr	125	0.5	0.83	4-10	132-173	5,460-7,038	11,838-17,565	103,873	M
	Blood, 1yr	125	0.5	0.92	4-10	133-173	5,461-7,037	11,911-17,615	120,853	M
	Blood, 1yr	100	0.5	0.83	4-10	132-173	5,156-6,736	12,140-17,872	90,419	M
	Blood, 1yr	100	0.5	0.92	4-10	133-173	5,157-6,735	12,213-17,921	105,405	M
	Blood, 1yr	75	0.4	0.74	5-11	130-170	4,909-6,524	12,109-17,866	136,665-234,399	S
	Blood, 1yr	75	0.4	0.83	5-10	131-171	4,911-6,515	12,207-17,932	67,410-196,646	S
	Blood, 1yr	75	0.5	0.74	4-10	132-172	4,853-6,440	12,359-18,119	98,363-162,016	S
	Blood, 1yr	50	0.3	0.83	5-11	128-169	4,688-6,322	12,154-17,867	149,042-285,889	S
	Blood, 1yr	50	0.3	0.92	5-11	129-169	4,688-6,319	12,230-17,928	133,653-232,493	S
	Blood, 1yr	25	0.3	0.74	5-11	128-168	4,372-6,005	12,399-18,107	104,606-241,807	S
	Blood, 2yrs	125	0.5	0.92	5-11	127-169	4,678-6,310	12,036-17,956	130,133-477,198	S
	Blood, 2yrs	100	0.5	0.83	5-11	125-168	4,469-6,112	12,074-18,040	119,698-1,913,392	S
	Blood, 2yrs	100	0.5	0.92	5-11	127-169	4,470-6,101	12,245-18,167	93,107-362,692	S
	Blood, 2yrs	75	0.5	0.74	5-11	124-166	4,259-5,903	12,165-18,109	105,937-2,186,623	S

Scenario Class	Test, interval	Test Cost[a]	Sensitivity[b]		CRC Deaths[c]	QALYG[c,d]	Costs[c,d]	NMB[e]	ICER[f]	
			AA	CRC						
	Blood, 2yrs	75	0.5	0.83	5-11	125-168	4,261-5,903	12,282-18,250	72,577-1,364,461	S
	Blood, 2yrs	75	0.5	0.92	5-11	127-169	4,262-5,893	12,454-18,377	56,081-255,456	S
	Blood, 2yrs	50	0.4	0.92	6-12	123-165	4,176-5,813	12,170-18,062	Reference strategy	S
	Blood, 2yrs	50	0.5	0.74	5-11	124-166	4,051-5,694	12,374-18,320	38,092-1,171,119	S
	Blood, 2yrs	50	0.5	0.83	5-11	125-168	4,053-5,694	12,491-18,461	25,456-815,531	S
	Blood, 2yrs	25	0.4	0.83	6-12	122-164	3,965-5,604	12,242-18,128	Reference strategy	S
	Blood, 2yrs	25	0.4	0.92	6-12	123-165	3,964-5,601	12,382-18,276	Reference strategy	S
	Blood, 2yrs	25	0.5	0.74	5-11	124-166	3,843-5,485	12,582-18,531	Reference strategy	S

Notes: CRC = Colorectal cancer, AA = Advanced adenoma, LYG = Life-years gained, QALYG = Quality-adjusted life-years gained, NMB = Net-monetary benefit, ICER = Incremental cost-effectiveness ratio.

a) CMS cost per screening test (applied to those above age 65). See Supplementary Table 2 for detailed costs.
b) Sensitivity of colonoscopy refers to lesion-level sensitivity, whereas sensitivity for non-invasive tests refers to “person-level sensitivity”, which considers the most-advanced lesion of a screened individual.
c) Outcomes presented as events or life-years per 1,000 45-year-olds.
d) Discounted at 3% per year.
e) Net monetary benefit (NMB) per person setting a willingness to pay of $100,000 per QALYG (i.e., NMB = 100,000 * QALYG – Net Costs). Net costs are the cost of the screening regimen minus the costs of the “No Screening” regimen. In this context, the net monetary benefit can be interpreted as the average absolute monetary value for those who adhere to a screening regimen, accounting for all benefits and costs incorporated in the analysis. Taking the difference between the NMB of two strategies reveal the loss of benefit caused by the choice of a sub-optimal strategy. For instance, a strategy that yields half of the NMB of the optimal strategy might be
f) Since No screening is a dominated strategy, ICER is not calculated for the least effective non-dominated strategy. M, C and S refers to MISCAN, CRCSPIN and SIMCRC, and are displayed if each of those models project the test to be cost-effective (i.e., strategy was not dominated and had an ICER lower than $150,000. ICER ranges reflect the range of ICERs for the models that projected a strategy not to be extended dominated.
g) ICER is not computed by any model because screening strategy is dominated.
h) The no Screening strategy has no test cost or sensitivity values.
i) Costs depend on findings at colonoscopy. See Supplementary Table 2.

References

1. Imperiale TF, Ransohoff DF, Itzkowitz SH, et al. Multitarget stool DNA testing for colorectal-cancer screening. *N Engl J Med*. Apr 3 2014;370(14):1287-97. doi:10.1056/NEJMoa1311194

2. Screening for Colorectal Cancer - Blood-Based Biomarker Tests. Accessed October 16, 2023. https://www.cms.gov/medicare-coverage-database/view/ncacal-decision-memo.aspx?proposed=Y&NCAId=299

3. Zhao S, Wang S, Pan P, et al. Magnitude, Risk Factors, and Factors Associated With Adenoma Miss Rate of Tandem Colonoscopy: A Systematic Review and Meta-analysis. *Gastroenterology*. May 2019;156(6):1661-1674.e11. doi:10.1053/j.gastro.2019.01.260

4. Ladabaum U, Mannalithara A, Brill JV, Levin Z, Bundorf KM. Contrasting Effectiveness and Cost-Effectiveness of Colorectal Cancer Screening Under Commercial Insurance vs. Medicare. *Am J Gastroenterol*. Dec 2018;113(12):1836-1847.

5. Find Lab Tests. Epi proColon®, Septin 9 Gene Methylation Detection in online lab tests stores. Accessed June 14, 2023. https://www.findlabtest.com/lab-test/general-wellness/epi-procolon-septin-9-gene-methylation-detection-labcorp-481160#:~:text=None%20Epi%20proColon%C2%AE%2C%20Septin,Blood%20Test)%20with%20price%20%24498.00.

6. Ness RM, Holmes AM, Klein R, Dittus R. Utility valuations for outcome states of colorectal cancer. *The American journal of gastroenterology*. 1999;94(6):1650-1657.
